# The Role of Preoperative Neutrophil, Platelet, and Monocyte to Lymphocyte Ratios as Independent Prognostic Factors for Patient Survival in WHO 2021 Glioblastoma: A Single-Center Retrospective Study

**DOI:** 10.7759/cureus.25801

**Published:** 2022-06-09

**Authors:** George S Stoyanov, Emran Lyutfi, Reneta Georgieva, Deyan L Dzhenkov, Lilyana Petkova, Borislav D Ivanov, Ara Kaprelyan, Peter Ghenev

**Affiliations:** 1 General and Clinical Pathology/Forensic Medicine and Deontology, Medical University of Varna, Varna, BGR; 2 Neurology and Neuroscience, Medical University of Varna, Varna, BGR; 3 Faculty of Medicine, Medical University of Varna, Varna, BGR; 4 Clinical Medical Science, Medical University of Varna, Varna, BGR

**Keywords:** survival, glioblastoma, monocyte to lymphocyte ratio, platelet to lymphocyte ratio, neutrophil to lymphocyte ratio

## Abstract

Introductions

Immuno-oncology is a rapidly developing field wherein tumor-immune system interactions can be harnessed for diagnostics. Herein, we set out to establish the role of the immune system response, as measured by preoperative neutrophil, platelet, and monocyte to lymphocyte ratios (NLR, PLR, and MLR) as prognostic markers for patient survival based on the newly defined criteria for glioblastoma (GBM).

Materials and methods

The study included patients diagnosed with GBM at a four-year interval. Exclusion criteria were patients subject to reoperation in the time period; tumors in more than one system; a history of hematological and autoimmune diseases; and cases with infectious or other inflammatory conditions. Data regarding patient demographics and preoperative blood counts were pulled from patient records and compared to postoperative survival.

Results

A total of 22 patients fit the established criteria, with a male to female ratio of 2.14:1, a mean age of 66.23 years, and a mean survival of 255.72 days (8.04 months, range 24-801 days). Eight patients had an elevation of NLR and five of PLR, with no statistical correlation to survival. Six patients had an increase in MLR with a statistically significant (p=0.0044) shorter postoperative survival. Synergic increases in NLR and PLR did not show significance, while synergic increases with MLR showed no added benefit.

Conclusion

Preoperative MLR, but not NLR or PLR, is a promising independent biomarker for patient survival in GBM. It is suggested that elevations in these ratios directly correlate to tumor biological potential.

## Introduction

Glioblastoma (GBM), as defined by the 2021 World Health Organization (WHO) classification of central nervous system (CNS) tumors, is an isocitrate dehydrogenase (IDH) wildtype and histone 3 (H3) wildtype tumor with predominantly astrocytic differentiation and at least one of the following: microvascular proliferation, necrosis, TERT promoter mutation, EGFR gene amplification, and +7/10 chromosome copy-number changes [1]. GBM is the most common malignant primary CNS tumor and is fast-growing and extremely aggressive, with a short postoperative survival time of little more than a year, according to most studies [2,3]. With the changes implemented in the 2021 WHO classification, GBM is a tumor characteristic of older males, previously classified as GBM tumors with IDH and H3 mutations, now falling into their own diagnostic units while also being characteristic of younger individuals, with IDH and H3-wildtype pediatric tumors separated into their own entity based on their genetic profile [1].

The practical implications of these WHO CNS classification changes will decrease the mean survival of GBM patients, as the newly separated nosological units have far better prognoses. Furthermore, as the changes in the classification are based on detectable mutations with prognostic significance, this leaves a few such established markers for GBM, namely O-6-methylguanine-DNA methyltransferase (MGMT) promoter methylation, and others in need of verification, as they were established based on cohorts now representative of mixed nosological units [4-6].

Despite its more than 100 years of practical application history, a rapidly developing field in oncology is immuno-oncology, wherein the tumor-immune system interactions have the potential to be harnessed for diagnostic, predictive, and treatment purposes [7,8]. These interactions are multifaceted and can be viewed as both local tumor-immune system reactions - e.g., tumor-infiltrating lymphocytes or a response of the immune system on a systemic level to the presence of malignant processes [8]. The systemic immune response can be measured using hematological biomarkers, namely neutrophil to lymphocyte (NLR), thrombocyte/platelet to lymphocyte (PLR), and monocyte to lymphocyte ratios (MLR), which reflect an abnormal state of the immune response [9-12]. Variance in these parameters has been established to have prognostic significance in multiple conditions ranging from inflammatory and traumatic to cardiovascular and malignant [12-14]. Herein, we set out to establish the role of NLR, PLR, and MLR as prognostic markers for patient survival based on the newly defined WHO CNS 2021 criteria for GBM [13-16].

## Materials and methods

A retrospective non-clinical approach was utilized for the means of the study. Patients with a histologically and molecular verified GBM, corresponding to the 2021 WHO CNS tumor classification guidelines, diagnosed in a four-year interval (February 2018 to February 2021) in a single tertiary healthcare institution were included. Exclusion criteria were other non-GBM CNS tumors, patients with an initial diagnosis before the specified time frame subject to reoperation, tumors in more than one system, history of hematological and autoimmune diseases, and cases where the preoperative consults established an infectious or another inflammatory disorder.

Patient demographics and preoperative blood counts of neutrophils, thrombocytes, monocytes, and lymphocytes were pulled from patient records and compared with postoperative patient survival.

Preoperative ratios were calculated as absolute numbers using the following formulas:

$NLR = \frac{neutrophils (10^{9}/l)}{lymphocytes (10^{9}/l)}$

$PLR = \frac{platelets (10^{9}/l)}{lymphocytes (10^{9}/l)}$

$MLR = \frac{monocytes (10^{9}/l)}{lymphocytes (10^{9}/l)}$

Statistical analysis

Results were statistically analyzed with MedCalc version 19.7.1 (MedCalc Software Ltd, Ostend, Belgium) using a descriptive statistical approach. Survival analysis was performed utilizing the Kaplan-Meier method with 95% confidence intervals; a p-value of <0.05 was considered significant. Kaplan-Meier groups were separated in a bimodal manner with preserved and elevated ratio groups, with generally accepted cutoff values as follows: >4 for NLR, >200 for PLR, and >0.25 for MLR, and compared to postoperative patient survival.

## Results

A total of 22 patients fit the established inclusion criteria without contradicting the exclusion ones, of whom 68.18% (n=15) were male and 31.82% (n=7) were female, a male to female ratio of 2.14:1. The mean age of the cohort was 66.23 years old (range 50-86 years old), with a mean age for males of 64.07 (range 50-85) and a mean age for females of 70.86 (range 52-86). The mean survival was 255.72 days (8.04 months, range 24-801 days).

The mean cell counts were as follows (in 10^9^/L): neutrophils - 15.81 (median 6.77, range 2.94-76.4); platelets - 284.1 (median 271.5, range 141-538); monocytes - 0.91 (0.64, range 0.27-6.6); lymphocytes - 2.06 (median 1.65, range 0.76-6.7). As seen by these figures, there is significant variance in all evaluated factors.

NLR

**Figure 1:**
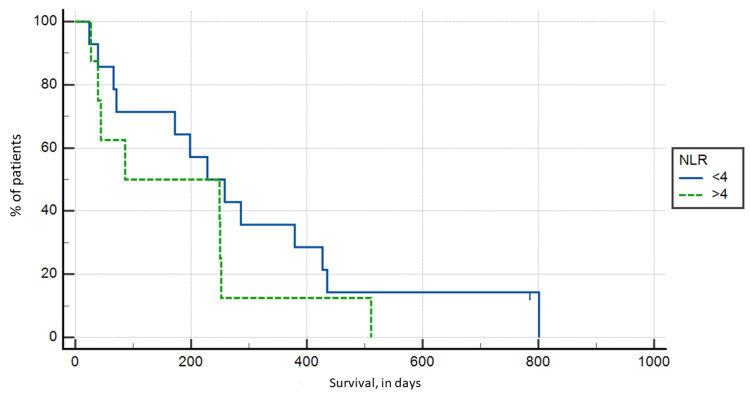
Kaplan-Meier survival analysis based on neutrophil to lymphocyte ratio groups

As already mentioned, the NLR cutoff value used was >4. A total of eight patients had an NLR > 4, with the highest ratio being 77.17, while the remaining 14 patients had an NLR of <4, with the lowest ratio being 1.81. The Kaplan-Meier survival analysis did not show statistical correlation (p > 0.05), with a mean of 182.25 days of postoperative survival for the NLR > 4 group, compared to 297.79 days in the NLR < 4 group (Figure 1).

PLR

**Figure 2:**
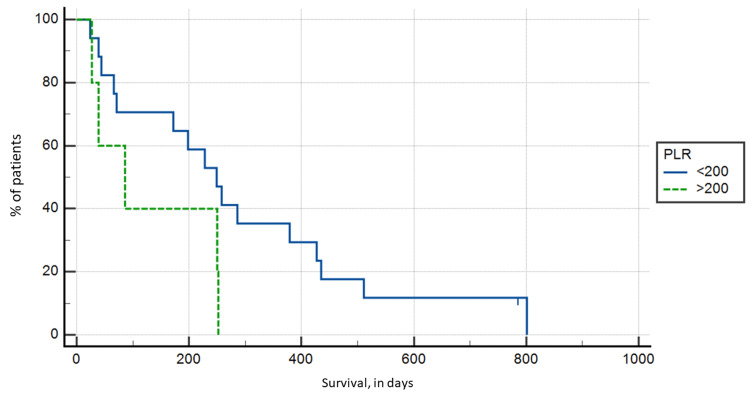
Kaplan-Meier survival analysis based on platelet to lymphocyte ratio groups

A total of five patients had a PLR > 200, with the highest ratio being 301.74, while the remaining 17 patients had a PLR < 200, with the lowest ratio being 43.73. The Kaplan-Meier survival analysis again showed no statistical significance in survival (p > 0.05), with mean survival in the PLR > 200 group of 130.8 days compared to 292.53 days in the PLR < 200 group (Figure 2).

MLR

**Figure 3:**
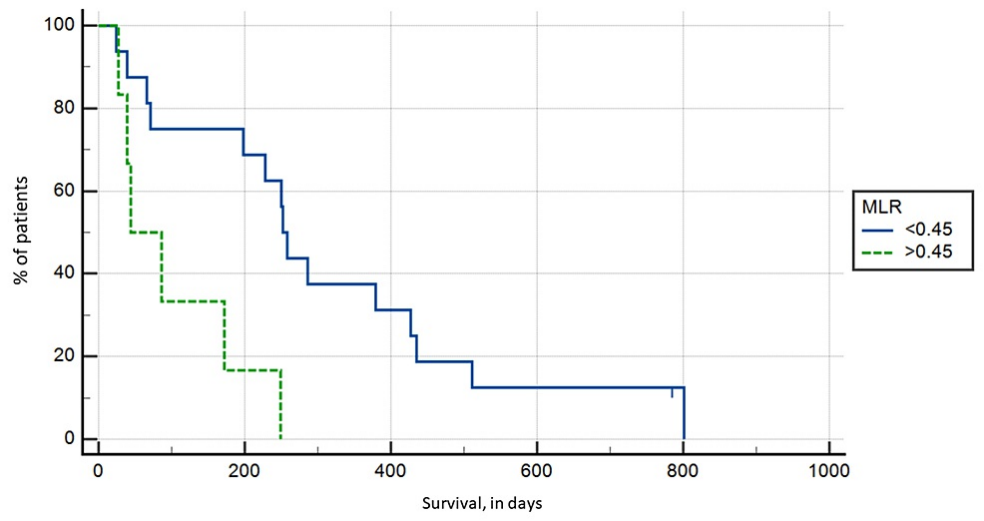
Kaplan-Meier survival analysis based on monocyte to lymphocyte ratio groups

A total of six patients had an MLR > 0.45, with the highest ratio being 4.07, while the remaining 16 patients had an MLR < 0.45, with the lowest ratio being 0.09. The Kaplan-Meier survival analysis showed a statistically significant (p = 0.0044) shorter postoperative survival in the MLR > 0.45 group with a mean survival of 103.83 days compared to 313.13 in the MLR < 0.45 group (Figure 3).

NLR, PLR, and MLR synergy

**Figure 4:**
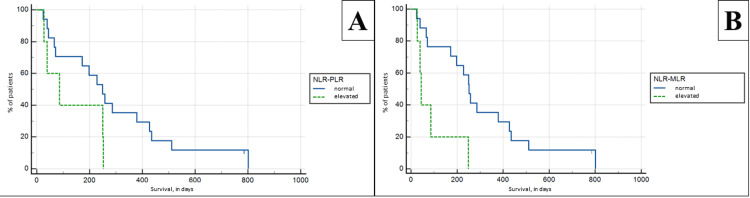
Kaplan-Meier survival analysis based on synergic neutrophil to lymphocyte and platelet to lymphocyte ratio (A) and neutrophil to lymphocyte and monocyte to lymphocyte ratio (B)

A synergic increase in NLR and PLR was seen in five patients, meaning that all patients with an elevated PLR also had an elevated NLR. As evident from the individual values of the ratio, there was no statistically significant difference in patient survival (p > 0.05), with a mean survival in the NLR-PLR group of 130.8 versus 292.53 days in the group with non-elevated values (Figure 4A).

A synergic increase in NLR and MLR was seen in five patients. The Kaplan-Meier survival analysis showed statistically significant lower survival, although the significance of this indicator was lower than MLR alone, p = 0.0062, and survival was 100.25 versus 291.56 days (Figure 4B).

**Figure 5:**
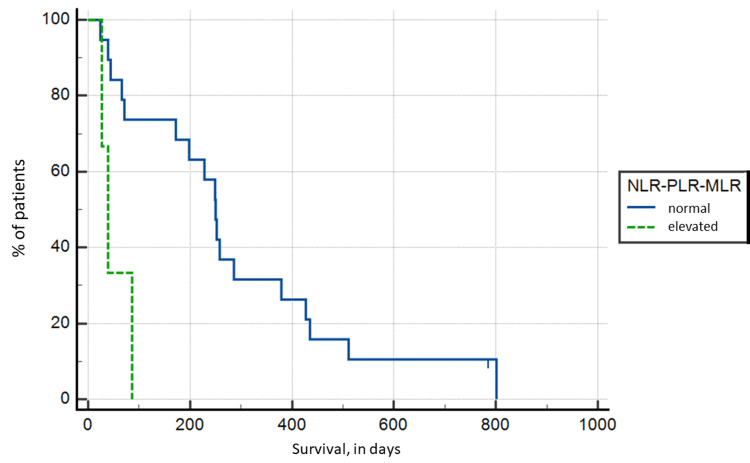
Kaplan-Meier survival analysis based on synergic neutrophil to lymphocyte, platelet to lymphocyte, and monocyte to lymphocyte ratio

A synergic increase in PLR and MLR was seen in three patients, with the patients also having an elevated NLR as well. The Kaplan-Meier survival analysis showed preservation of the statistical power of MLR alone with p = 0.0044, and survival of 50.67 versus 289 days for the group with non-elevated combined indices (Figure 5).

Correlation with MGMT promoter methylation

From the 22 patients included in the study, a total of eight had MGMT promoter methylation, an independent predictive factor for temozolomide treatment response. Only two of the patients with MGMT promoter methylation had an increase in the studied factors: one with an elevated NLR ratio of 43.62 and postoperative survival of 511 days (16.79 months), the other with a synergic increase in NLR of 77.17 and PLR of 222.22 and postoperative survival of 250 days (8.21 months). These data suggest that NLR, PLR, and MLR elevation occur predominantly in GBM without MGMT promoter methylation and, if present in patients with MGMT promoter methylated GBM, does not interfere with patient prognosis. However, no added benefit was established statistically when comparing the ratios to patient survival in non-MGMT promoter-methylated GBM.

## Discussion

The state of the systemic immune response, as measured by us using the elevated levels of NLR, PLR, and MLR in terms of tumor localization in the CNS, has the potential to be a promising biomarker. This is primarily due to the lack of contact of the CNS with the external environment and the low native incidence of inflammation, sporadic or tumor-associated, before surgical intervention when compared to other tumor sites - lung, urinary system, colon, etc. [9,17-19].

Regarding the state of the systemic immune response in recent years, the scientific literature has accumulated much data on the importance of preoperative NLR for patient survival, with some authors reporting statistical significance not only for survival in GBM but also for tumor grade and as a distinguisher from CNS metastasis (Table 1) [10-12,20-27]. Interesting additional data from these studies are those of Weng and Kemerdere, where a positive correlation was found between the rate of increase in NLR and the histological grade of tumors, as well as data from Haksoyler, who proved that the prognostic value of the index is preserved even in disease recurrence [21,24,26]. One contradictory but promising result is the reported elevation of NLR by Figueroa in GBM patients undergoing laser interstitial thermal therapy, where the patients are reported to have a better outcome [20]. There are less data on the significance of the PLR, with conflicting reports on its relevance. While some authors report a statistically significant decrease in survival with the elevation of PLR and its role in distinguishing from lower-grade gliomas and metastasis, others, as in our cohort, report no such correlation [11,12,23,25-27]. An interesting fact that complements the yet unspecified nature of the PLR ​​index is that authors studying the combined role of NLR and PLR depict that the combined increase in ratios has higher statistical significance than either index alone, a correlation that, although without statistical significance, is valid in our sample as well [11,26]. Even fewer data exist in the literature on the MLR index, where, as in our sample, the most significant statistical significance in relation to shorter postoperative survival is found as a stand-alone marker and in combination with other markers of the inflammatory response [10,25,26]. However, these studies focus predominantly on the specificity of the marker for GBM in distinguishing it from other tumors [10,25,26].

**Table 1:** Comparative literature review table on relative published data regarding the role of NLR, PLR and NLR in GBM diagnosis and prognosis GBM: glioblastoma; WHO: World Health Organization; NLR: neutrophil to lymphocyte ratio; PLR: platelet to lymphocyte ratio; MLR: monocyte to lymphocyte ratio; LMR: lymphocyte to monocyte ratio

Study	Variable analyzed	Result	Added benefit
Subeikshanan et al. [10]	Peripheral blood biomarkers – cell counts and ratios in control and brain tumors, including intra and extra-axial tumors	GBM patients have elevated NLR compared to controls and decreased MLR	Ratios are preserved in non-malignant (extra-axial) tumors. Patients with GBM exhibit thrombocytosis. Monocyte infiltration causes a reduction in circulating monocytes.
Kaya et al. [11]	NLR and PLR in patients with GBM	GBM patients with elevated NLR have a statistically shorter postoperative survival of 11.8 ± 4.7 months versus 15.7 ± 2.5, p= 0.048. PLR not correlated with survival p=0.854	Combined elevation in NLR and PLR yields higher statistical significance for shorter survival - 11.8 ± 4.7 months versus 16 ± 2.2, p=0.026
Wang et al. [12]	NLR and PLR in patients with WHO grade I-IV glioma	Patients with elevated ratios have statistically significant shorter survival: NLR 20.75±7.68 months versus 26.91±6.91, p=0.001; PLR 21.61±6.35 months versus 25.53±8.62 months, p = 0.007	NLR statistically increases with WHO grade (p=0.001), while PLR does follow this tendency without statistical significance (p=0.055). Multivariate Cox regression identified only NLR ≥ 4.00 as an independent prognostic factor.
Figueroa et al. 2020 [20]	NLR in patients with GBM undergoing laser interstitial thermal therapy	Preoperative NLR did not affect survival (p=0.5204), while postoperative NLR increase had significance for improved survival (p=0.011)	The more significant the increase in NLR after the procedure, the better the prognosis
Haksoyler et al. [21]	NLR in patients with recurrent GBM treated with bevacizumab plus irinotecan	Low pre-initiation NLR yields a statistically better prognosis of 15.8 versus 9.3 months, p=0.015	Focuses exclusively on recurrent GBM
Lei et al. [22]	Metaanalysis on NLR in GBM	High NLR pre-operatively yields statistically shorter survival, p<0.00001; however, this is specific in Asians and not Caucasians. Elevation in NLE is not only significant for shorter survival in GBM but all gliomas	Meta-analysis of all relevant published data to that point
Lopes et al. [23]	Peripheral blood biomarkers – cell counts, NLR and PLR	NLR, PLR and cell counts of neutrophils, lymphocytes and platelets do not correlate with overall survival	Preoperative NLR ≤ 5 correlated with shorter progression-free survival. A subgroup analysis of patients with Stupp protocol showed that preoperative NLR > 7 correlates with shorter overall survival, p=0.023
Weng et al. [24]	NLR in patients with WHO grade I-IV glioma	In GBM with elevated NLR, postoperative survival is decreased - 11.23 months versus 18.56 months, p<0.05	NLR increases with WHO grade, p<0.05. NLR 2.36 is a cutoff for predicting glioblastoma.
Baran et al. [25]	Peripheral blood biomarkers – cell counts, NLR, PLR, and LMR in regards to tumor histogenesis	NLR, PLR and LMR show a different pattern in GBM compared to metastasis. LMR is the shows highest statistical significance in predicting GBM compared to metastasis, p=0.03	NLR and PLR are statistically higher in metastasis, p=0.05 and 0=004, respectively, and LMR decreases, p=0.01.
Kemerdere et al. [26]	NLR, PLR and LMR in patients with WHO grade I-IV glioma	NLR and PLR increase and LMR decrease correlate statistically with glioma grade, p=0.00001; 0.02 and 0.02, respectively	An increase in NLR is statistically a strong predictor for high-grade glioma, followed by LMR and PLR, p=0.00001; 0.004, and 0.009, respectively
Yang et al. [27]	Meta-analysis on peripheral blood biomarkers – cell counts, NLR and PLR in GBM	An increase in NLR is associated with worse postoperative survival, p=0.0007. Elevation in PLR shows no such correlation	Meta-analysis of all relevant published data to that point

An interesting correlation with the result is that most of the tumors we studied do not have MGMT promoter methylation in increased indexes. MGMT is part of the DNA damage repair system and is unique in regards to being a monoenzymatic system, unlike the other systems in DNA damage repair, such as mismatch repair [28]. Tumors deficient in MGMT are more susceptible to DNA damage, which is utilized in GBM treatment, in which deficient tumors are more susceptible to the temozolomide-related response. However, sporadic DNA damage in these tumors may induce necrosis, necroptosis, or pyroptosis, releasing cytoplasmic components into the bloodstream [29-30].

Such phenomena correlate with elevated serum levels of GFAP - the main cytoplasmic protein in glioma cells, tumor-related mRNA, tumor microvesicles and exosomes, and whole circulating tumor cells [30]. The fact that the newly defined 2021 WHO CNS tumor classification GBM is only an IDH-wildtype tumor further underlines the tumor's aggressiveness [1]. IDH-wild-type tumors, when compared to the IDH mutant forms from the 2016 WHO CNS tumor classification (now defined as astrocytoma, WHO CNS grade 4), show more extensive necrosis not only in the form of the pathognomonic pseudopalisadic - primary Scherer figures but also tumor (coagulative/ischemic) necrosis [1]. These extensive areas of necrosis would lead to accelerated release of cellular fragments, including inflammatory mediators and hence the immune system's response, with probably a higher amount of shed cellular particles correlating to a more intense response [29,30]. Thus, the established correlation with the immune system response directly reflects the cellular phenomena of tumor aggressiveness. As such inflammatory mediators released by the necrotic cells would yield an increase in peripheral neutrophils and hence reduce circulating lymphocytes, while tumor recruitment of peripheral monocytes will decrease their circulating number and stimulate the production and maturation of lymphocytes [10,15].

These suggestions are supported by the elevation of these indexes in a number of conditions in and outside of the CNS, ranging from inflammatory, degenerative, malignant, vascular, and traumatic, in all of which index elevation correlates with poor prognosis [13,14,16].

Study limitations and future directions

The main limitations of the current study are the small size of the studied cohort and the short survival period observed. As can be seen from the descriptive characteristics of the studied sample, it includes a small number of cases, with the number of patients with elevation in the studied indexes being even smaller. Future studies will need to have larger samples of homogenous tumors according to the WHO CNS tumor classification and estimate the significance of these indexes as reported by other authors and ourselves, and monitor any dynamic in regard to treatment response, disease recurrence, and progression. Furthermore, as most elevated indexes occur in unmethylated GBM variants, a correlation with molecular data on MGMT methylation for possible cutoff limit verification and tumor biological behavior should be encouraged.

## Conclusions

From the peripheral blood biomarkers we studied, only elevation in MLR showed statistical significance regarding postoperative patient survival in histologically and mutationally verified GBM. While other studies report significance also for NLR and PLR in glioblastoma and the correlation between the two, we were unable to confirm these results in our cohort statistically. These indexes seem to directly correspond with tumor aggressiveness and intratumoral cellular instability, as they are a reaction of the immune system to cellular debris shedding from tumor-associated necrosis. As evident from their association with poor prognosis in multiple other conditions with varying etiopathogenesis, they indicate a body-wine reaction to deviation from the norm.

Despite our small sample size, the most statistically significant ratio seems to be MLR. Future studies are encouraged to not only study larger populations but also monitor their ratios dynamically, not only to reproduce and verify or dispute our results but also to see if the dynamics of these biomarkers can be used, not only as a predictor for survival but also for disease recurrence and progression. Furthermore, as the new 2021 WHO CNS tumor classification has introduced significant changes to taxonomy, it would be encouraged to also study these factors in the newly separated entities as well as in a single group with different diagnostic approaches - histological (classical) GBM versus molecularly verified GBM with a lower histological grade.
